# Development of an Intentional Telehealth Viewing Guide for Home-Based Patient Assessment

**DOI:** 10.1089/tmr.2020.0017

**Published:** 2021-01-22

**Authors:** Kimberly D. Shea, Victoria Towers, Melissa Koon, Graciela Silva

**Affiliations:** ^1^ College of Nursing, University of Arizona, Tucson, Arizona, USA.; ^2^ Casa de la Luz Hospice and Palliative Care, Tucson, Arizona, USA.

**Keywords:** best practice, health care, quantitative content analysis, home assessment, telehealth, televideo

## Abstract

**Background:**

The increased use of telehealth to visit patients in their home permits greater access to care, and also increases the opportunity for whole-person assessments that improve individualized care. The videoconferencing camera is a proxy for home visit provider's eyes. However, cameras limit views, thereby reducing environmental cues. The Novice to Expert Theory of skill acquisition supports the use of an intentional viewing guide to assure a comprehensive patient assessment using telehealth in the home (CPATH). This study advances the development of a CPATH framework to guide providers to be intentional when using televideo technology.

**Methods:**

A quantitative content validity approach was used to determine the validity of *a priori* items within domains that were in the original protocol framework. A content validity determination requires 5–10 experts to rate agreement (range 1–5) on items within domains. Our sample was composed of seven expert home health providers. More than five experts had to agree to achieve statistical significance (*p* < 0.05) for validity.

**Results:**

Of the 15 items in the protocol, only 8 items had significant agreement for the sample size. These items were breathing, nonverbal gesturing, positioning, oxygen, safety, and types, dosages, and administration guidance of medication. Other items were added within the existing domains of Patient Characteristics, Treatment and Equipment Functioning, Medications and Environmental Quality, with the exception of Caregivers.

**Conclusion:**

The domains triggered considerations for existing or additional items that require assessment, thereby developing the intentional guide framework that permits individualization of a telehealth home-based visit.

## Introduction

Telehealth is the use of technology to deliver health care services at a distance by using telecommunication technology.^1^ The current coronavirus disease 2019 (COVID-19) pandemic has advanced telehealth more in a few months than researchers, health care providers, technology companies, and other telehealth advocates have in 20 years. Telehealth use has burgeoned. From April 2019 to April 2020, there was an 8,336% increase in telehealth insurance claim lines.^2^ Over 70% of primary care providers now conduct visits with patients in their homes using telehealth technology.^3^ However, despite the recent surge in remote technology-assisted home visits, visiting patients in their homes is as old as caring.

The formal practice of health-related home visits in the United States was for conditions of illness, poverty, or maternal and infant care.^4^ Home-based medical care continued until the end of the 1700s when institutionalized health care formalized payment for delivery of services. Following the small pox pandemic of 1880, transmission of disease was of great concern, so the *Visiting Nurse Service* became the model for house calls. In the last 15–20 years, house calls, with the use of technology, by nurse practitioners and primary care physicians, have made a comeback.^5^ In 2020, as in 1880, contagious conditions have influenced a historic change as to where and how patients receive care.

In the Institute of Medicine's report, *Crossing the Quality Chasm*, six aims for the public and private health care systems define care.^6^ Care is to be safe, effective, patient-centered, timely, efficient, and equitable. With these aims in mind, telehealth technology can be applied to how patients receive care at home. However, receiving care from providers in a patient's home, as opposed to in a health care facility, also requires consideration of self-management abilities. The ability to manage without the constant oversight of a provider is the tipping point for remaining in the home. Knight and Shea's Empowerment Informatics Framework elaborates on the use of technology to empower patients with self-management as a primary contributor.^7^ Careful and intentional assessment of what is available in the home and possible for the patients and family is critical for success.

An assessment of a patient's condition can be superficial, if the lifestyle, environment, and support system are not included. Telehealth is today's answer to keeping patients safe and providing quality care for those most vulnerable to disease. However, relatively speaking, we are all novices in the use of televideoconferencing and other remote telehealth technologies.

Telehealth use is a new skill that requires guidance. The use of trial and error novice learning is a barrier to quality patient care. With this in mind, consulting experienced home care providers is a crucial first step in the development of a viewing guide when using video technology to capture what is important for a comprehensive home-based assessment. The purpose of this research was to develop a framework to guide providers to conduct a comprehensive patient assessment using telehealth in the home (CPATH) through the application of televideo technology.

## Background

Home health care nurses are examples of comprehensive observers who utilize a whole-person approach to the provision of patient-centric care. When entering a home, certain cues alert these keen observers, such as problems that may exist with the care of the patient, environmental factors that may contribute to the sickness of a patient, and missing items that enable the patient to thrive. Items such as throw rugs that may cause tripping, medications being stored in a haphazard manner, pain that is exacerbated by the bedding, mold, or other allergens growing, or a chair leg sitting on an oxygen tube are only a few of the infinite cues that can be observed while in the home. Other examples are sparse food in the refrigerator that may indicate that there is a need for additional resource management; or the living style of others in the home that may be dangerous to the patient.

Specifically, homebound patients are vulnerable to many different environmental concerns because they are often bedbound and totally dependent on others for daily tasks and decision-making. Therefore, comprehensive observations, typically ascertained during in-person visits, help the provider to detect the need for additional services, medications, equipment, or education that would benefit the caregiver and the patient. However, with the growing development and utilization of technology, mobile device cameras can be used as a proxy for the eye of the observer to conduct similar assessment from afar.

Use of mobile devices for social visits is common to people of all ages, even older adults. It is noted that 77% of adults over the age of 65 years own a cell phone, and 59% have access to the internet.^8^ Because of the COVID-19 pandemic, these statistics have likely increased and facilitated the increased use of mobile devices for the provision of health care. Mobile devices in conjunction with providers using secure software programs enable patients to remain in their homes without the concern for risking their privacy or confidentiality.

Easy access to care provides the convenient benefits of reduction in travel time to appointments, rapid visual assessments by the nurse or health care provider, follow-up care as needed, and psychological support for the patient and caregivers in their homes. However, challenges do exist with technology use, necessitating different assessment techniques by providers. Use of mobile devices that are familiar to the patient improves ease of use; however, these devices often provide a restricted view from the device's narrow-angle camera lens, stationary positioning, and an interrupted assessment (if there is low bandwidth for either patient or provider).

A comprehensive whole-person assessment is a process that incorporates the patient's concerns, providers' knowledge, and in-the-moment cognizance of items that further inform the assessment. The technology and the lack of cues from the environment of an in-person, hands-on assessment present challenges that change the process during a remote visit. Preparation before the visit supplements these challenges.

An intentional process, specifically for remote visits, in contrast to the nurse or health care provider being able to observe arbitrarily, improves the efficiency and effectiveness of the visit. Therefore, as health care moves into an era in which telehealth becomes common, an opportunity arises to embrace the best of the in-person home visit, enabling consideration of the true essence of the patient and adding the benefits of technology. For novice telehealth providers, a guide for intentional remote visits is necessary to acquire the skill of telehealth visits. For expert telehealth providers, the guide will serve as an internalized framework to use best practices intuitively for remote assessments.

## Theory

The study is guided by the Novice to Expert framework for skill acquisition.^9,10^ A skill is simply defined as the ability to do something well.^11^ By practicing a learned foundation, skills, such as providing a telehealth home visit, will progress appropriately toward expertise. Novices use guides as the foundation of learning how to perform a skill in the best way possible. However, guides are integral throughout the trajectory from novice to expert.

The five stages of the framework are as follows: novice user, advanced beginning user, competent user, proficient user, and expert exemplify different characteristics benefiting from a telehealth guide. The novice user focuses on succeeding and guides to direct each step in the skill. The advanced beginning user starts to troubleshoot using learned knowledge and information and the guide content to better understand the context of the home assessment. The competent user solves problems using the guide to select what is relevant. The proficient user looks at the bigger picture having internalized the content of the guide. Ultimately, the expert uses the internalized guide to be a source of knowledge to teach and inform others. Given the novelty, of using mobile devices for home videoconferencing, telehealth users are novices.

The challenges that the technology presents are with respect to care that is possible when the expert can provide a “hands-on” assessment and home evaluation in-person. Therefore, in this study, we learned from these experts about what content to include in the development and validation of a guide for a CPATH.

### Study design

The study design consisted of a quantitative content validity approach by Lynn to determine the validity of items within domains in the CPATH framework.^12^ There are two types of validity: face and content. In-person home assessments are based on assumptions of what is relevant to evaluate when with a patient. Face validity is associated with assumptions and definitions as determined by experts. Content validity, used in this study, determines statistically significant agreement among experts of relevance of items in a data collection instrument.

The CPATH is essentially an instrument to guide the collection of information during a remote home visit, and therefore, the use of content validity is an applicable approach. The first stage of Lynn's^10^ quantification of content validity is the Development Stage. During this preparatory stage, researchers (with home health background) identified domains, generated items, and compiled a list of potential items for assessment.^10^ The second stage, Judgment and Quantification, requires data collection from experts and statistical analysis of their data.

### Setting and sample

Following approval by human subjects from the institutional review board, a convenience sample was recruited from a large palliative and hospice agency (>400 average daily census) in southwestern Arizona. The home care program utilizes nurse case managers, nurse practitioners, and medical doctors to assess seriously ill patients. Study researchers presented the protocol to these home care experts at a monthly meeting. Criteria for inclusion in the study as an expert were that the case managers had clinical expertise and had provided home visits for at least 1 year.

### Data collection

**FIG. 1.**
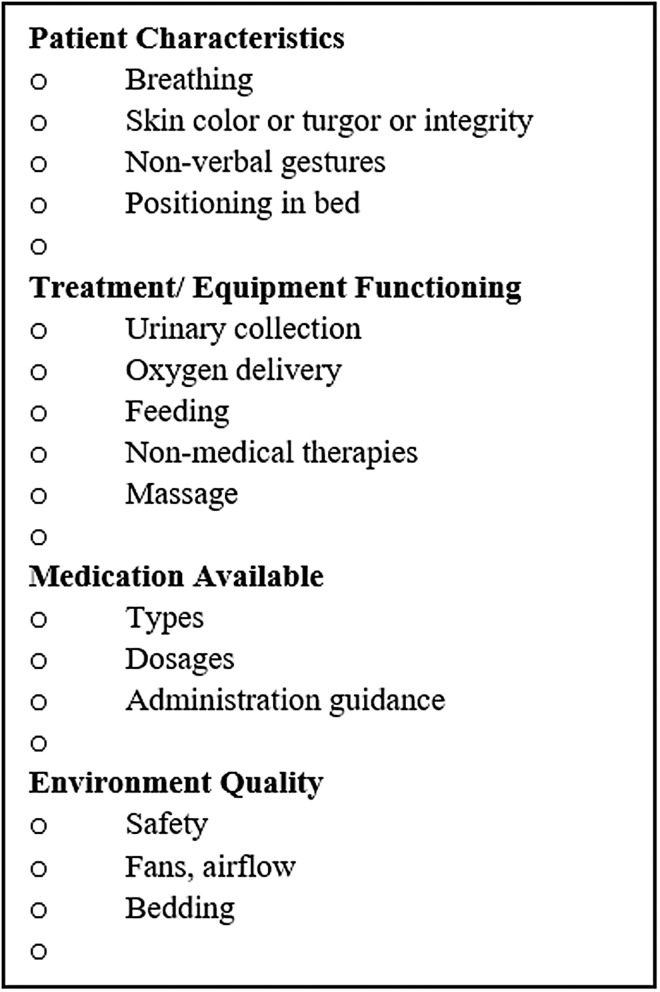
Original items for experts to rate by a 5-point scale (strongly disagree to strongly agree) and additional items.

Based on face validity from the authors' experiences in home visits, a list of 15 items within four domains (*Patient Characteristics*, *Treatment and Equipment Functioning*, *Medications*, and *Environmental Quality*) were developed *a priori* (Fig. 1). The list containing items within domains was given to the experts who consented to participate. The following instructions were provided:

“Envision that you are entering a patient's home for a nursing visit. Think about what you would look at to evaluate the situation. Then look at the items in the list. Next to each item rate how strongly you agree with the need to view each item when you go into a home. To rate the items use a 5-point scale with, 5 = strongly agree and 1 = strongly disagree. After you rate the items then please add additional items that you feel are missing from the list.” The participants submitted their rating of each item and comments into a sealed box at the conclusion of the meeting.

### Analysis

Determination of the validity of the content for a home assessment is crucial because the researchers needed to be confident that the list would accurately guide users to assess patients in their home. The ranked data for each item (1–5), as evaluated by the experts, were entered into an Excel spreadsheet for analysis. A 5-point scale provided greater sensitivity for the analysis of variance in the rankings. Based on Lynn's statistical formula,^10^ the number of experts required to agree (agree or strongly agree) on an item is dependent on the number of experts participating.^2^ For an item to be retained in the guide, agreement that the item was necessary with less than a 5% chance of error (*p* < 0.05) is required. The standard deviation (SD) then determines the highest to lowest ranking items. Items that had the lowest variation in rankings indicated that there was the strongest agreement among the experts. Items not receiving the minimum agreement of experts or ranked low were eliminated from the list, resulting in the final CPATH framework.

## Results

Seven nurse case managers with a minimum of 1-year experience consented to participate in the study and completed the process of assessing the content validity of the *a priori* items. Using the content validity process, when there are seven participating experts, six or seven are required to agree to obtain *p* ≤ 0.05. Table 1 displays that all the participants were middle-aged females having experience in home care of >7 years. All were positive proponents of telehealth use for care and five of seven were eager to know when the agency might start this type of care.

**Table 1.** Demographics

Demographics of sample (n = 7)	
Profession	7—Registered Nurses
Age	1—30–40 years old
	5—40–50 years old
	1—50+ years old
Gender	7—female
Years of experience in home care visits	1—3–7 years
	6—7–16 years
Attitude toward remote visits with camera	7—positive
	5—asking when it will be available for use

Table 2 displays the results of the content validity analysis. Of the 15 items in the protocol, only eight items had significant agreement from six or more experts. These items *were breathing*, *nonverbal gesturing*, *positioning*, *oxygen*, *safety*, and *types*, *dosages*, and *administration guidance of medications*. Three items were ranked as the most needed items to evaluate *nonverbal physical indicators or gestures*, *medication administration guidance,* and *safety* (*p* ≤ 0.05, SD = 0.0). Three items were ranked as the second-most needed items to evaluate *breathing*, and *types* and *dosages of medications* (*p* ≤ 0.05, SD = 0.38). *Positioning* (*p* ≤ 0.05, SD = 0.49) was ranked third and *oxygen* (*p* ≤ 0.05, SD = 0.76) was fourth. Seven items were not significant (*p* > 0.05), and therefore have a potential for elimination from the telehealth assessment guide list. These were *skin-color*, *turgor or integrity*, *urine collection*, *feeding*, *nonmedical therapies*, *massage*, *fans-airflow* and *bedding*.

**Table 2.** Results of Content Analysis

Assessment areas of concern	p ≤ 0.05	SD	Rank
Physical characteristics			
Breathing	X	0.38	2
Skin		1.06	8
Nonverbal gestures	X	0	1
Positioning	X	0.49	3
Treatment equipment functioning			
Urine collection		0.79	5
O_2_	X	0.76	4
Feeding		0.95	6
Nonmedical		0.79	5
Massage		1.35	
Medications available			
Types	X	0.38	2
Dosage	X	0.38	2
Administrative guide	X	0	1
Environmental quality			
Fans		1.0	7
Safety	X	0	1
Bedding		1.7	9

SD = standard deviation.

Three of the seven experts commented that viewing the caregiver needed to be added. Two experts added nebulizers or continuous positive airway pressure (CPAP) machines. Other single items noted for addition were *walker*, *wheelchair*, *transfer equipment*, *environmental clutter*, *wounds and edema*, *side rails*, *“chux” (disposable underpad)*, *and positioning of the head of the bed*. In total, 53% of the items were significantly valid to be retained in the guide. To double-check the outcomes of the content validity process, the rating scale was transformed into a 3-point Likert scale (agree, neutral, and disagree). The results did not change.

## Discussion

**FIG. 2.**
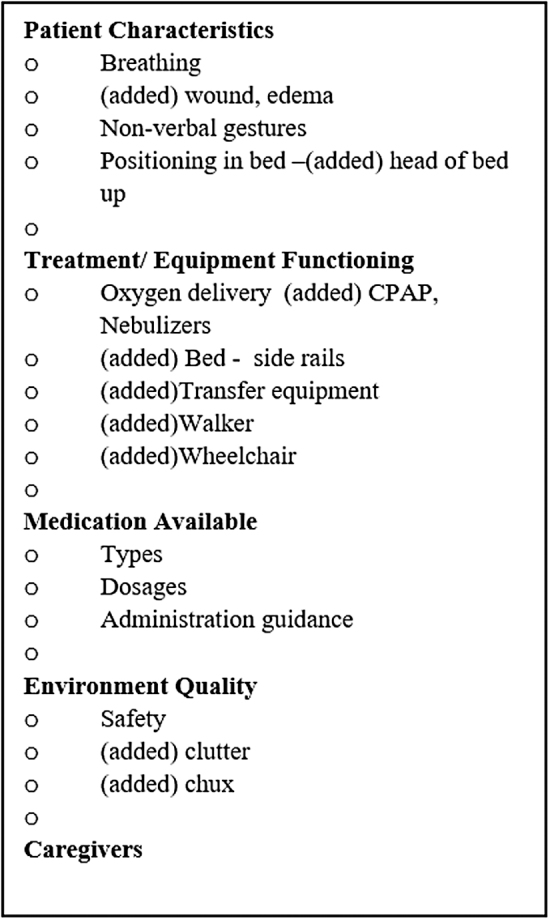
Resulting validated items and including (added) items. CPAP = continuous positive airway pressure.

The *Medication* domain had the greatest number of highest rankings and agreement on items, with all items to be retained in the guide. The *Physical Characteristics* domain would retain three items, with only *skin* at risk for elimination. *Treatment and Equipment Functioning* and *Environmental Quality* each would retain a single item. By retaining the validated items and including expert added items, Figure 2 displays a newer version to compare with Figure 1. While the added items have not been validated, examining where they would be located within the existing domains is revealing. The validated items are ubiquitous to every patient, regardless of diagnosis and situation, while the added items are those that are individualized.

Domains, while part of the process in the development stage, took on greater meaning following data analysis. When comparing Figures 1 and 2, the domains ultimately triggered considerations of which items were most necessary to view. Domains were specific enough to prompt thoughts of diagnosis and situation-related concerns and broad enough to be inclusive of all that make a person whole. Almost every item added by an expert could be included as an item within one of the four domains, except caregiver. Caregivers are a determining factor in whether patients can remain at home, in most situations. The omission of Caregiver as a domain is an unfortunate initial oversight and undoubtedly needs to be added as a fifth domain.

All of the home care experts were experienced in working with seriously ill patients; therefore, they viewed the content of the home assessment guide with respect to that type of patient. Before COVID-19, the majority of home visits were for seriously ill patients who were not well enough to leave their homes. Now visits using telehealth in the patient's home have become common for many diagnoses. Telehealth home visits will most likely remain for patients who are vulnerable to infectious viruses and at risk for death. Using the domains alone can serve as a quick prompt for planning a CPATH. The CPATH guide could be individualized through using domains to incorporate what is already known about the patient (i.e., diagnosis, risks), with observational cues enabling real-time health care interventions. One final concern resulting from the data analysis is that nonmedical items remained the lowest ranking items, casting some doubt on the perceived value of potential alternative options for assessment during telehealth visits.

## Conclusion

The majority of today's health care interactions between providers and patients are limited by the providers' time availability. Telehealth and in-person visits both run the risk of providers trying to engage in a hastened review of the patient in an effort to streamline a visit and remain on schedule. However, when in the home, even the subtlest of cues can help the provider to move toward a whole comprehensive patient assessment. As stated earlier, limited camera views reduce access to such cues, and if the provider is additionally distracted by the need to rush through a patient review during a telehealth visit, the assessment becomes a one-sided perception of who and what the whole person is. This sets up an enormous risk that providers may miss important information and arrive at incomplete assessments, which can lead to inaccurate clinical decisions.

Having a guide with established domains to prompt providers to intentionally view aspects of the patient's home during a telehealth visit advances the opportunity for providers to better understand the total patient's health and safety needs. Based upon the review by expert nurses, there was strong agreement that *medication type*, *dosage and administration*, *nonverbal gestures*, *breathing*, *oxygen*, *and safety* are crucial to be assessed during a home visit. However, other items were added that the expert nurses believed to be necessary for the patient that they were considering. The other items mostly fit within the domains of *Treatment and Equipment Functioning* and *Environmental Quality*, with the additional suggestion to include assessing the *Caregiver* as a fifth domain.

The domains triggered considerations for an individualized telehealth home visit. Using domains as a guide, coupled with the importance of ensuring that any home visit conducted via telehealth maintains a patient-centered focus, providers will be able to complete an intentional comprehensive assessment of the patient using the CPATH.

## Future Work

The emerging CPATH guide with five domains is currently being trialed in a seriously ill patient sample to determine if intentional viewing using the domains and items, agreed upon by the experts, is acceptable to patients and caregivers. This mixed-methods study allows the patient and caregivers to express their perspectives on using a camera to assess the whole person. Domains as a prompt for primary care telehealth visit preparation, which will assure mobile camera technology is used efficiently and intentionally, will be explored in future research. Finally, this study focused on the perspective of the expert health care provider.

## Abbreviations Used

COVID-19: coronavirus disease 2019
CPATH: comprehensive patient assessment using telehealth in the home
SD: standard deviation
